# Bilateral scaphoid fractures: Case report and literature review

**DOI:** 10.1016/j.ijscr.2020.01.005

**Published:** 2020-01-14

**Authors:** Abdullah E. Kattan, Mohammed A. Almarghoub, Nujaim H. Alnujaim, Omar Barasain, Noor H. AlLababidi

**Affiliations:** a Division of Plastic and Hand Surgery, King Saud University, Riyadh, Saudi Arabia; b Plastic and Reconstructive Surgery Section, Department of Surgery, King Faisal Specialist Hospital & Research Center, Riyadh, Saudi Arabia; c College of Medicine, Imam Abdulrahman University, Dammam, Saudi Arabia

**Keywords:** Scaphoid fracture, Mechanism of injury, Bilateral wrist pain, Road traffic accident

## Abstract

**Background:**

Traumatic bilateral scaphoid fractures are extremely rare. Here, we report an unusual mechanism of bilateral scaphoid fracture after road traffic accident.

**Case report:**

A 32-year-old male presented with bilateral wrist pain to our plastic surgery clinic six months after he was involved in a road traffic accident. He was diagnosed with bilateral scaphoid fracture based on his CT scan findings. It is noteworthy that at the time of collision, he had grasped the steering wheel strongly to brace himself and had suffered no significant injuries except for bilateral wrist pain.

**Discussion:**

The diagnosis may be missed in our case because of bilateral hand pain complaints and normal X-ray report. Thus, we suggest that CT scan should be performed in similar incidents, particularly in case of normal X-ray reports.

**Conclusion:**

It is recommended that CT scan should be performed in similar mechanism of injury with a high index of suspicion.

## Highlights

• Scaphoid is the most common carpal bone involved in fractures.
• Presentation of bilateral scaphoid fracture is extremely rare.
• Bilateral scaphoid fractures rarely occur after RTA.
• High index of suspicion and good clinical judgment are required to detect bilateral scaphoid fractures.

## 1 Introduction

Scaphoid is the most common carpal bone involved in fractures and commonly occurs as unilateral traumatic fractures in males as a result of low-energy falls on the hands [1]. It is estimated that scaphoid fractures comprise 2.4% of all wrist fractures in the United States and has an incidence of 1.47 fractures per 100,000 person years [2]. In addition, the incidence of scaphoid fractures is higher in males in comparison to females [1,3]. Acute presentation within the first week of injury helps in its early management, and any delay in treatment results in surgical intervention and functional limitation [4]. A high index of suspicion, X-rays and CT scans are required for early diagnosis and management of scaphoid fractures to avoid complications such as osteoarthritis, chronic pain, decreased range of motion, etc. Scaphoid bone fractures are commonly caused due to falls on outstretched hands and are rarely observed post road traffic accidents (RTA) [4,5]. A study conducted in a trauma unit in the United Kingdom between 2007 and 2008 reported that only 3.3% of scaphoid fractures were caused by RTAs [1]. Scaphoid fracture is generally reported in one hand, and the presentation of bilateral scaphoid fracture is extremely rare [6]. The latter occurs as stress fractures due to repetitive stress on the wrists from sports activities [7]. In this report, we present an uncommon mechanism of bilateral scaphoid fracture after RTA. To the best of our knowledge, there are currently no similar reported cases in the literature. We found only one case with similar mechanism of injury, but the patient was riding a motor bike [5]. This work has been reported in line with the SCARE criteria [8].

## 2 Case report

A 32-year-old male presented with bilateral wrist pain that persisted for the last 6 months along with limitation in his work and daily activities. He was a smoker and had no medical or surgical history of significance. Six months ago, the patient was involved in RTA. Prior to this accident, the patient had a healthy condition with no pain at both wrists. At the time of collision, he was driving the car at a speed of 80–90 km/h and had not fastened his seat belt. He did not face any roll over or ejection from the car. The patient had no previous trauma or accident before the RTA.

**Fig. 1:**
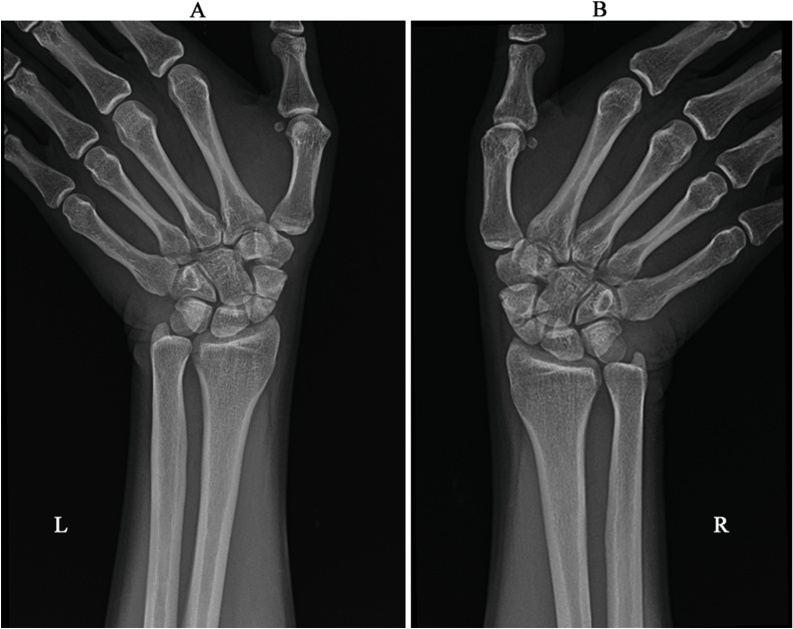
X-rays showing anteroposterior views with ulnar deviation of both writs showing bilateral scaphoid fractures.

**Fig. 2:**
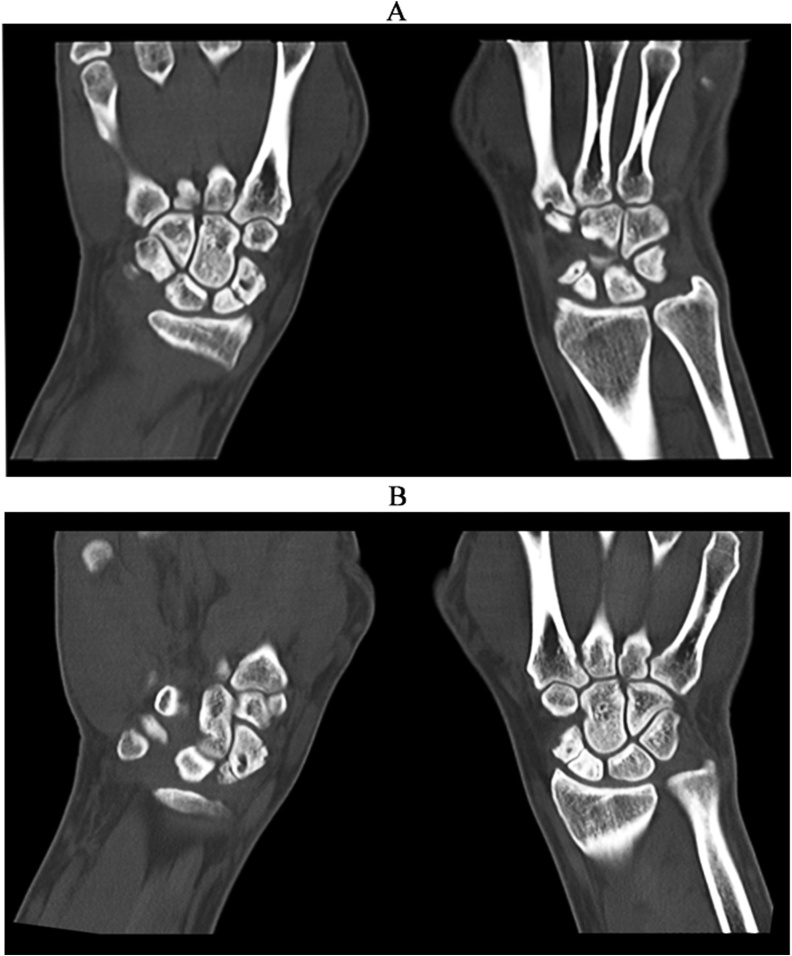
CT scan showing coronal views with bilateral scaphoid fractures.

The incident occurred suddenly when the car in front of him suddenly stopped, and he had grasped the steering wheel strongly to brace himself. He felt pain immediately after the accident in both hands and thus visited the emergency room. No significant injuries were reported at that time, and his X-rays were within normal. He was discharged the same day. It should be noted that no CT scan was done for the hands at that time. Six months later, the patient presented to our plastic surgery clinic with bilateral hand pain, more in the left wrist. The patient reported that he was functioning normally with little pain but no limitation in range of motion for the past four months. But the pain gradually increased such that it affected his daily work and activity. On examination, he was vitally stable and had an unremarkable general examination. Hands examination showed no swelling nor redness, and the neurovascular status was intact. He had bilateral tenderness on anatomical snuff box and minimal restriction of movement on flexion and pronation of the wrists. His X-rays showed bilateral scaphoid fractures (Fig. 1), and the diagnosis was confirmed by CT scan (Fig. 2).

## 3 Discussion

Scaphoid acts as a link between proximal and distal carpal rows and transfers the load from the hand to the forearm. It is essential for maintaining wrist stability. Scaphoid is the most commonly fractured carpal bone and accounts for 2.4% of all wrist fractures [2]. Patients with scaphoid fractures present with a history of wrist hyperextension beyond 95 degrees, i.e., falling onto an outstretched hand [5]. Moreover, a prospective study found that 3.3% of scaphoid fractures are caused due to RTAs [1].

In 80% of scaphoid fractures, the waist of scaphoid is fractured [9]. This is because the distal pole moves freely, and the proximal pole of scaphoid is trapped between capitate, radius and palmar capsule [9]. Moreover, unilateral scaphoid fracture is a well-established presentation, while bilateral scaphoid fracture is a less common entity [6]. In general, bilateral scaphoid fracture occurs as stress fractures in competitive athletes participating in sports requiring repetitive stress on the wrists in extension position [4]. It is generally observed in gymnasts, goalkeepers, and divers who present with chronic wrist pain without an acute injury in their teenage years [5].

Our aim in this report is to describe a unique mechanism of injury that caused a traumatic bilateral scaphoid fracture. In our case, the patient presented with bilateral wrist pain after an RTA during which he had grasped the steering wheel to brace himself. He visited the emergency room, where his X-rays were found to be normal and he was not investigated thoroughly. Six months later, the patient presented again to the clinic with increased pain and functional limitation, and bilateral anatomical snuff box tenderness. His X-rays and CT scan showed bilateral scaphoid fractures. In the literature, we found a similar mechanism of injury, but the patient was riding a motorcycle, unlike our patient who was driving a car [5]. In that patient, the fracture was discovered in the emergency room immediately after the accident, but in our case, the diagnosis was missed and discovered six months after the accident. Both patients suffered RTAs by colliding with decelerating cars in front of them. Both patients had both wrists in extension while grasping the handles/steering wheel at the time of collision. Both patients also presented with tenderness over the anatomical snuffbox. The diagnosis may be missed in our case because of bilateral hand pain complaints and normal X-ray report. Thus, we suggest that CT scan should be performed in similar incidents, particularly in case of normal X-ray reports.

The main limitation of our study is the uncertainty of causes of scaphoid fractures as our patient could have had other injuries after the accident. However, the patient emphasized that he had no other injuries in the time period between RTA and presentation to our clinic.

A high index of suspicion and good clinical judgment are required to assess patients presenting with subtle injuries of the wrist to exclude scaphoid fractures. Early detection helps in the prevention of late complications of undetected scaphoid fractures such as non-union, secondary surgical interventions, osteoarthritis and wrist stiffness, etc. Since scaphoid fractures are commonly missed initially, our case and similar cases in the literature suggest that the best option for managing patients with suspected scaphoid fractures is to perform an early CT scan to detect the fracture. Future studies are encouraged to estimate the incidence of bilateral scaphoid fractures with RTAs.

## 4 Conclusion

CT scan should be performed in similar mechanism of injury with a high index of suspicion.

## Sources of funding

Nil.

## Ethical approval

The study was approved by King Saud University, College of Medicine Ethical review board committee.

## Consent

Informed consent was obtained from the participant included in this study.

## Author contribution

Nujaim H. Alnujaim, Omar Barasain Noor and H. AlLababidi collected the data.

Mohammed Almarghoub and Abdullah E. Kattan diagnosed and followed the patient.

## Registration of research studies

Nil.

## Guarantor

Mohammed A. Almarghoub.

## Provenance and peer review

Not commissioned, externally peer-reviewed.

## Declaration of Competing Interest

Nil.
